# Lrig1 marks a population of gastric epithelial cells capable of long-term tissue maintenance and growth *in vitro*

**DOI:** 10.1038/s41598-018-33578-6

**Published:** 2018-10-15

**Authors:** Pawel J. Schweiger, Ditte L. Clement, Mahalia E. Page, Troels Schepeler, Xiangang Zou, Gabor Sirokmány, Fiona M. Watt, Kim B. Jensen

**Affiliations:** 1 BRIC - Biotech Research & Innovation Centre, University of Copenhagen, DK-2200 Copenhagen N, Denmark; 2 Wellcome Trust - Medical Research Council Cambridge Stem Cell Institute, Tennis Court Road, Cambridge, CB2 1QR UK; 3 Centre for Stem Cells and Regenerative Medicine, King’s College London, 28th floor, Tower Wing Guy’s Campus, London, SE1 9RT UK; 4 Cancer Research UK Cambridge Institute, CB2 0RE Cambridge, UK; 5 Novo Nordisk Foundation Center for Stem Cell Biology, Faculty of Health and Medical Sciences, University of Copenhagen, DK-2200 Copenhagen N, Denmark; 6 Present Address: Department of Physiology, Semmelweis University, Tűzoltó street 37-47 1094, Budapest, Hungary

## Abstract

The processes involved in renewal of the epithelium that lines the mouse stomach remain unclear. Apart from the cells in the isthmus, several other populations located deeper in the gastric glands have been suggested to contribute to the maintenance of the gastric epithelium. Here, we reveal that Lrig1 is expressed in the basal layer of the forestomach and the lower part of glands in the corpus and pylorus. In the glandular epithelium of the stomach, Lrig1 marks a heterogeneous population comprising mainly non-proliferative cells. Yet, fate-mapping experiments using a knock-in mouse line expressing Cre specifically in Lrig1^+^ cells demonstrate that these cells are able to contribute to the long-term maintenance of the gastric epithelium. Moreover, when cultured *in vitro*, cells expressing high level of Lrig1 have much higher organoid forming potential than the corresponding cellular populations expressing lower levels of Lrig1. Taken together, these observations show that Lrig1 is expressed primarily by differentiated cells, but that these cells can be recruited to contribute to the maintenance of the gastric epithelium. This confirms previous observations that cells located in the lower segments of gastric glands can participate in tissue replenishment.

## Introduction

The stomach is an important part of the gastrointestinal tract with multiple functions. Serving as a reservoir for ingested material, it can expand and contract upon requirement. The peristaltic movements governed by the muscles surrounding the stomach ensure that the content is mixed thoroughly, while enzyme and acid secretion initiates the first stages of the digestive process before material is released into the small intestine. The epithelial surface facing the lumen is responsible for conditioning of the content and is compartmentalised into pits and glands to provide specific functions. The human stomach consists exclusively of glandular epithelium that can be divided into two primary sections the corpus and pylorus, which are responsible for acidification and enzyme secretion, respectively. In contrast, the upper part of the mouse stomach, the forestomach, consists of a stratified epithelium similar to the epithelium found in the oesophagus^1^. The epithelium that lines the entire stomach lumen undergoes constant replenishment in order to maintain long-term tissue function. Different mechanisms have been proposed for maintenance of the glandular gastric epithelium, with the dominant model suggesting that cells located in the isthmus of glands are the major source for tissue replenishment^2^. However, studies using long-term lineage tracing experiments have demonstrated that cells located outside of the isthmus region in both the corpus and pylorus contribute to tissue renewal^3,4^.

The negative regulator of ErbB signalling, Leucine Rich Repeats And Immunoglobulin Like Domains 1 (Lrig1), was initially identified as a marker of stem cells in the epidermis^5,6^. Subsequent work demonstrated that it is also expressed by stem cells in the small intestine and colonic epithelium^7,8^. Initial observations suggested that Lrig1 marked quiescent stem cells in intestinal and colonic epithelium^7^, Lrig1-expressing cells have subsequently been shown to be highly proliferative and contribute actively to the maintenance of the epidermis, small intestine and colon^8,9^. Numerous studies of cancerous tissues have indicated changes in the expression of Lrig1 upon malignant progression^10^, suggesting that Lrig1 might be a universal marker of cells with potential to contribute to the maintenance of adult tissues. Faithful models for monitoring the behaviour of Lrig1-expressing cells are required in order to conclusively address this hypothesis.

Somatic stem cells have the capacity to self-renew and give rise to differentiated progeny. This makes them essential for long-term tissue maintenance, and various assays have been developed to assess stem cell potential both *in vitro* and *in vivo*. Fate mapping based on irreversible labelling of specific cell populations with a genetic mark is the golden standard for monitoring *in vivo* stem cell potential. The most widely accepted technique relies on the expression of Cre recombinase fused to the modified version of the oestrogen receptor ligand-binding domain under the control of specific promoters. This provides temporal and spatial control of Cre-mediated recombination upon treatment with oestrogen analogous, such as tamoxifen. Upon activation, the recombinase mediates excision of gene elements flanked by *loxP* target sequences. This allows irreversible labelling of cells with e.g. fluorescent proteins following elimination of a *loxP*-flanked STOP cassette. It is subsequently possible to follow the behaviour of the labelled cell and its progeny over time^11^. As an alternative to *in vivo* assays, it is now possible to culture stem cells under conditions that maintain their self-renewal and differentiation potential^12^. It is consequently possible to assess the stem cell potential of particular subsets of cells in essentially any tissue.

Using a recently developed mouse model, which enables faithful monitoring of Lrig1-expressing cells, we provide evidence that Lrig1 marks subsets of cells in all parts of the stomach. In the forestomach as well as the glands of the gastric glandular epithelium, the progeny of these cells are maintained long-term and show contribution to the replenishment of the stomach epithelium. Although only a subset of the Lrig1-expressing cells contributes to long-term tissue maintenance, a large proportion of these cells have *in vitro* stem cell potential. We conclude that Lrig1 is expressed by a heterogeneous population of cells in all parts of the stomach epithelium and that some of them can be recruited to become stem cells.

## Results

### Generation of an Lrig1 reporter mouse model

**Figure 1:**
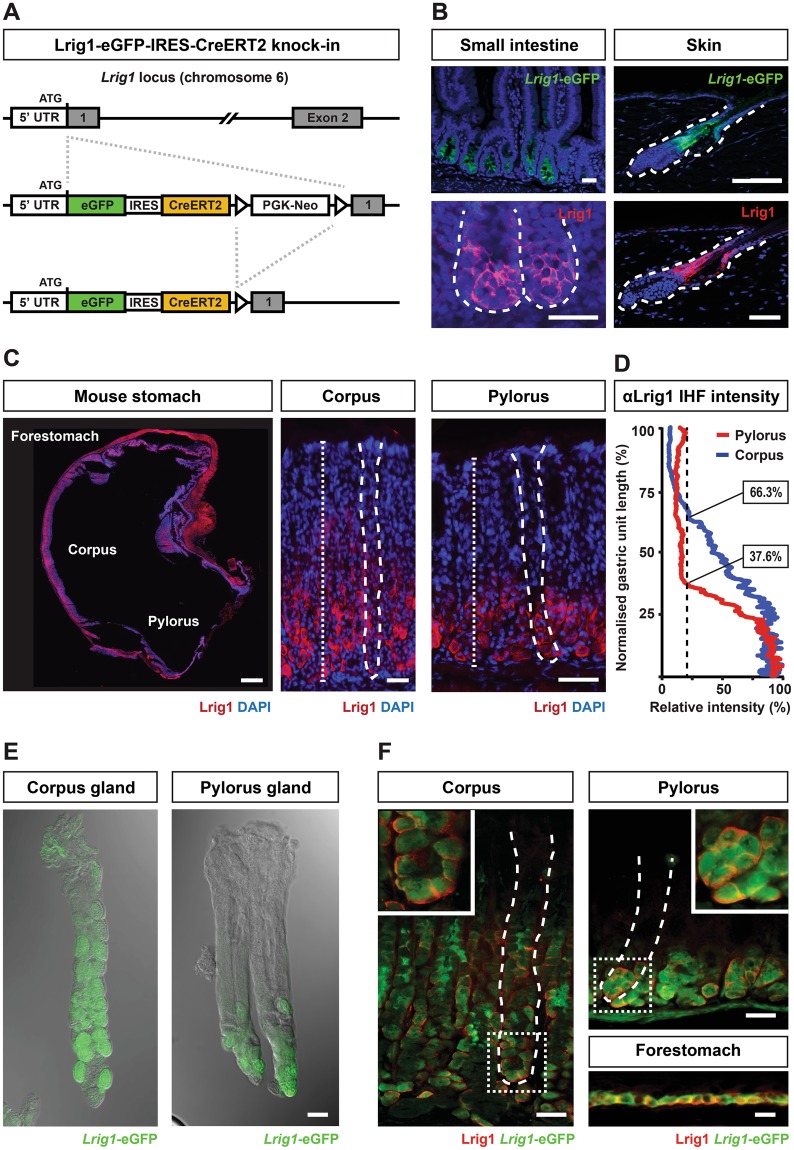
Generation of a reporter Lrig1 knock-in mouse line. (**A**) Schematic representation of the targeting strategy used to generate the Lrig1 knock-in mouse model. An eGFP-IRES-CreERT2 expression cassette was integrated into exon 1 of the endogenous *Lrig1* locus using two homology arms. PGK-Neo – neomycin resistance cassette; 5′ UTR – 5′ untranslated region; 1 – protein-coding region of the first exon of the *Lrig1* gene. (**B**) Expression of Lrig1 and eGFP in the epidermis and small intestine in the knock-in mouse model. (**C**) Expression of Lrig1 in different regions of mouse stomach. Vertical dotted lines represent examples of paths used to quantify Lrig1 expression. (**D**) Quantitation of Lrig1 immunohistofluorescence signal. Red and blue lines represent averaged intensity profile measured along the axes of gastric glands. Dotted line represents staining background cut-off (**E**) Expression of *Lrig1*-eGFP visualised as endogenous fluorescence in gastric glands isolated from corpus and pylorus of Lrig1 knock-in mouse model. (**F**) Co-expression of *Lrig1*-eGFP and Lrig1 in three stomach compartments in the Lrig1 knock-in mouse model: forestomach, corpus and pylorus. Scale bars: (**B**) −10 μm (small intestine), 30 μm (skin); (**C**,**E**,**F**) −25 μm.

In order to address the role of Lrig1-expressing cells in multiple tissues and perform a detailed characterisation of these cells, we used an Lrig1 knock-in mouse model. This mouse model allowed us to readily identify cells with an active Lrig1 promoter, and to assess the behaviour of these cells *in vivo*. The translational start site in exon 1 of the endogenous Lrig1 locus was targeted by homologous recombination in embryonic stem cells with a cassette encoding eGFP-IRES-CreER^T2^ (Lrig1KI; Fig. 1A). As reported previously, Lrig1 was expressed in multiple tissues including the epithelium of the skin^9^ and the intestine^8^. In line with the published patterns of Lrig1 expression, eGFP was detected robustly in the expected cell populations within the junctional zone of the pilosebaceous unit in the skin epidermis as well as at the bottom of crypts of Lieberkühn in the small intestine and colon (Fig. 1B). Based on the observed fluorescence pattern of eGFP, we conclude that the Lrig1 KI mouse model faithfully reports endogenous Lrig1 expression.

### Distinct populations of cells in the stomach epithelium express Lrig1

It has been demonstrated that Lrig1-expressing epithelial cells in the skin and the intestine contribute to long-term tissue homeostasis^7,9^. Previously published expression data indicated that Lrig1 was expressed in additional tissues including the stomach^13,14^. Analysis of endogenous Lrig1 revealed its robust expression in gastric units below the isthmus often concentrated in the lower neck and at the base in both the corpus and pylorus (Fig. 1C,D). Therefore, we sought to investigate the role of Lrig1-expressing cells in the three parts of the stomach epithelium: the forestomach, the corpus and the pylorus.

Analysis of the Lrig1 KI mouse model revealed that native eGFP could be detected at the bottom of glands isolated from both the corpus and the pylorus (Fig. 1E). Here, it was evident that GFP was expressed by discrete subsets of cells located at the bottom of the glands. The eGFP expressing cells were positive for Lrig1 confirming that the reporter mouse model faithfully reflected Lrig1 expression within the glandular epithelium (Fig. 1F). In contrast to the restricted expression in the glandular epithelium, Lrig1 was ubiquitously expressed in the basal layer of the forestomach.

In conclusion, Lrig1 was expressed by cells in the three parts of the stomach, but showed specific regional patterns that further substantiate the differences between these different tissue parts.

### Lrig1 marks a heterogeneous cell population in the glandular epithelium

**Figure 2:**
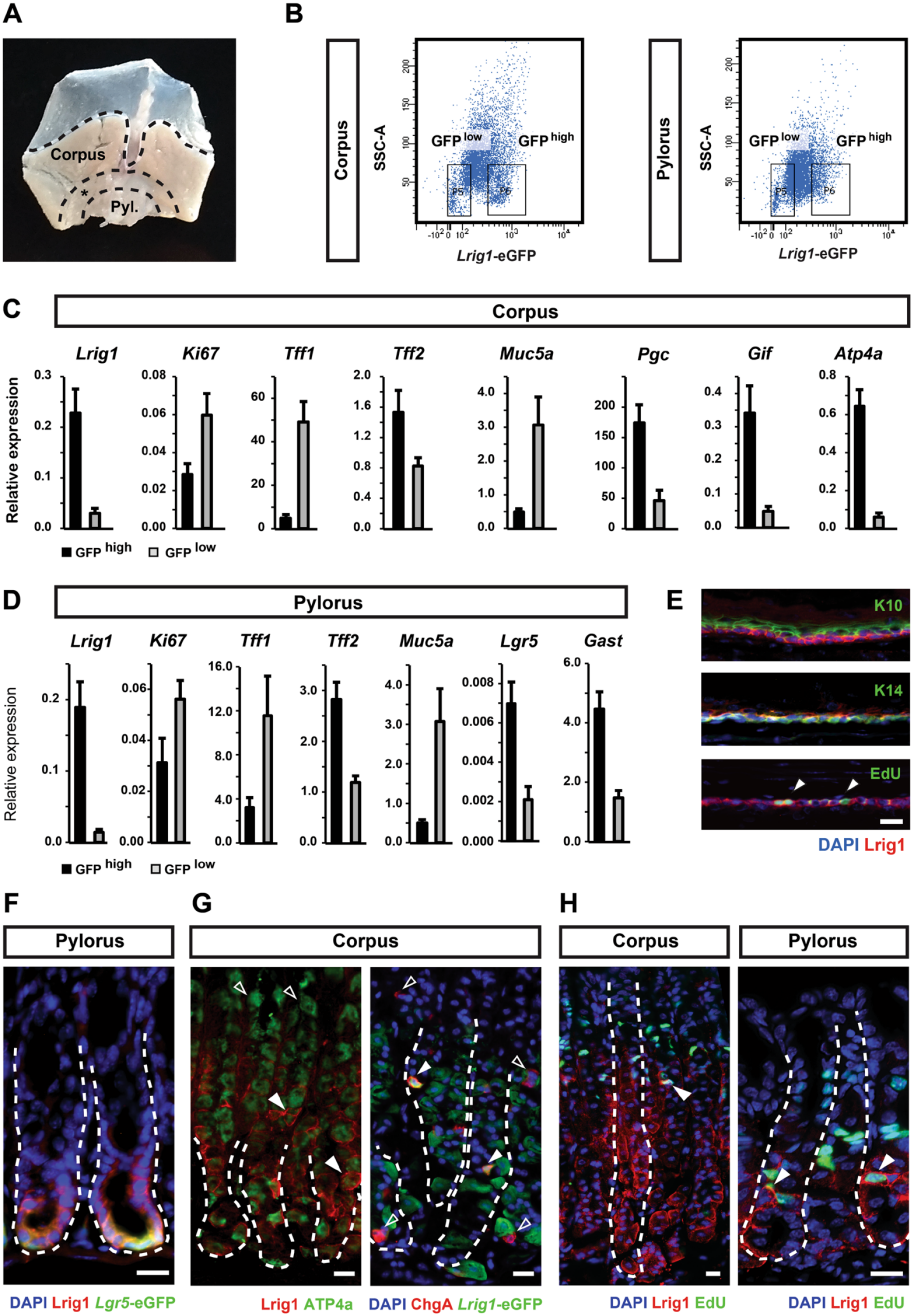
Characterisation of Lrig1-expressing cells in gastric epithelium. (**A**) Overview of the dissection strategy used to isolate corpus and pylorus from mouse stomach. The indicated margin between the two regions was discarded in order to avoid cross-contamination between compartments. (**B**) Flow cytometry analysis of mouse stomach epithelium showing differences in expression of *Lrig1*-eGFP and the separation strategy for FACS isolation of eGFP^low^ and eGFP^high^ cells. (**C**,**D**) qPCR gene expression analysis epithelial cells isolated by FACS from corpus and pylorus showing enrichment of different lineage markers in analysed cell populations. Expression levels are relative to *GAPDH*. (**E**) Expression of Lrig1 in the basal layer of mouse forestomach epithelium, showing overlapping pattern with keratin 14 (K14) and proliferating cells marked by EdU, but not keratin 10 (K10). (**F**) Co-localisation of Lrig1 and *Lgr5*-eGFP in the glandular epithelium of the pylorus. (**G**) Co-expression of stomach cell lineage markers, H+/K+ ATPase (Atp4a) and chromogranin A (ChgA) with Lrig1. (**H**) Co-localisation of Lrig1 expression with actively replicating visualised by staining of incorporated EdU. White arrowheads in (**G**,**H**) indicate cells co-localisation of markers with Lrig1. Empty arrowheads point to cells showing expression of a marker without simultaneous co-expression of Lrig1. Scale bars: E −25 μm, (**F**–**H**) −50 μm. *P ≤ 0.05.

The bottom of the gastric glands is characterised by the presence of a number of different cell types involved in the conditioning of the local microenvironment. This includes acidification by H^+^/K^+^ ATPase positive cells and secretion of various factors important for optimal digestion. Amongst cells located at the bottom are Lgr5- and Troy-expressing cells in the corpus, which both have the capacity to contribute towards the long-term maintenance of the epithelium^3,4^. In order to characterise Lrig1-expressing cells in the stomach, we isolated single cells by flow cytometry from dissected stomach corpus and pylorus (Fig. 2A) and assessed expression of key marker genes. Micro-dissection of the stomach tissue eliminating a wide margin of tissue between the corpus and the pylorus allowed for isolation of pure subsets of cells without cells from the transitional zone that separates the two parts. Using fluorescence-activated cell sorting of cells isolated from dissected mouse corpus and pylorus, we obtained two populations of epithelial cells expressing different levels of *Lrig1*-eGFP (i.e. *Lrig1*-GFP^high^ and *Lrig1*-GFP^low^ cells) (Figs 2B and S1). qPCR analysis of isolated cells confirmed the enrichment of *Lrig1* in the respective populations (Fig. 2C,D). Moreover, analysis revealed distinct patterns of marker expression between *Lrig1*-GFP^low^ and *Lrig1*-GFP^high^ cells. *Lrig1*-GFP^high^ cells in the corpus were enriched in markers for parietal cells (*ATP4a*), zymogenic chief cells (*Gif*) and mucous neck cells (*Pgc, Tff2*). At the same time, *Lrig1*-GFP^high^ cells were depleted for markers identifying surface mucous cell (*Muc5a* and *Tff1*) (Fig. 2C). This further substantiates the observation that Lrig1 was expressed by cells in the lower part of gastric glands, where parietal and chief cells were primarily found within the corpus. *Lrig1*-GFP^high^ cells isolated from the pylorus displayed elevated levels of *Lgr5* and the enteroendocrine marker *Gast* (Fig. 2D), both of which were expressed at the bottom of the pyloric glands. *Lrig1*-GFP^high^ cells from both corpus and pylorus expressed relatively low levels of Ki67.

### Lrig1 marks all proliferating cells in the forestomach, but not in the glandular epithelium

Analysis of marker expression on the population basis suggested significant heterogeneity within the Lrig1^+^ expressing population. In order to further characterise this population, we analysed the co-expression of established markers of the stomach epithelium at the single cell level.

Expression of Lrig1 in the forestomach was distinct from the expression in the glandular epithelium. In the stratified epithelium of the forestomach, Lrig1 was ubiquitously expressed by all cells in the keratin 14 positive basal layer including all the proliferating cells responsible for the maintenance of the epithelium (Fig. 2E). Within the glandular epithelium, the expression of Lrig1 extended beyond the region expressing *Lgr5*-eGFP^3^ (Fig. 2F). Importantly, meta-analysis of published expression data coalesced with these observations, since *Lrig1* was transcriptionally enriched in both the Troy (1.5 fold) and Lgr5 (2.8 fold) expressing populations^3,4^. Within the Lrig1-expressing population in the gastric epithelium, a large subset of cells was positive for ATP4a and a small population co-expressed chromogranin A (ChgA), whereas the remaining populations were intercalated between these cells (Fig. 2G). In addition, very few Lrig1-expressing cells were found to incorporate EdU following a short trace demonstrating that these cells are generally not proliferative (Fig. 2H). Consistent with the results of Ki67 expression analysis, this observation indicated that only a small fraction of the Lrig1-expressing cells in the glandular epithelium is proliferating. This aligns very well with the observation that Lrig1 is expressed primarily by differentiated non-proliferative parietal cells as well as chief cells that have been shown previously upon injury to constitute a reserve stem cell repertoire in the glandular epithelium.

### Lrig1-expressing cells in all parts of the stomach have the capacity to contribute long term to tissue maintenance

**Figure 3:**
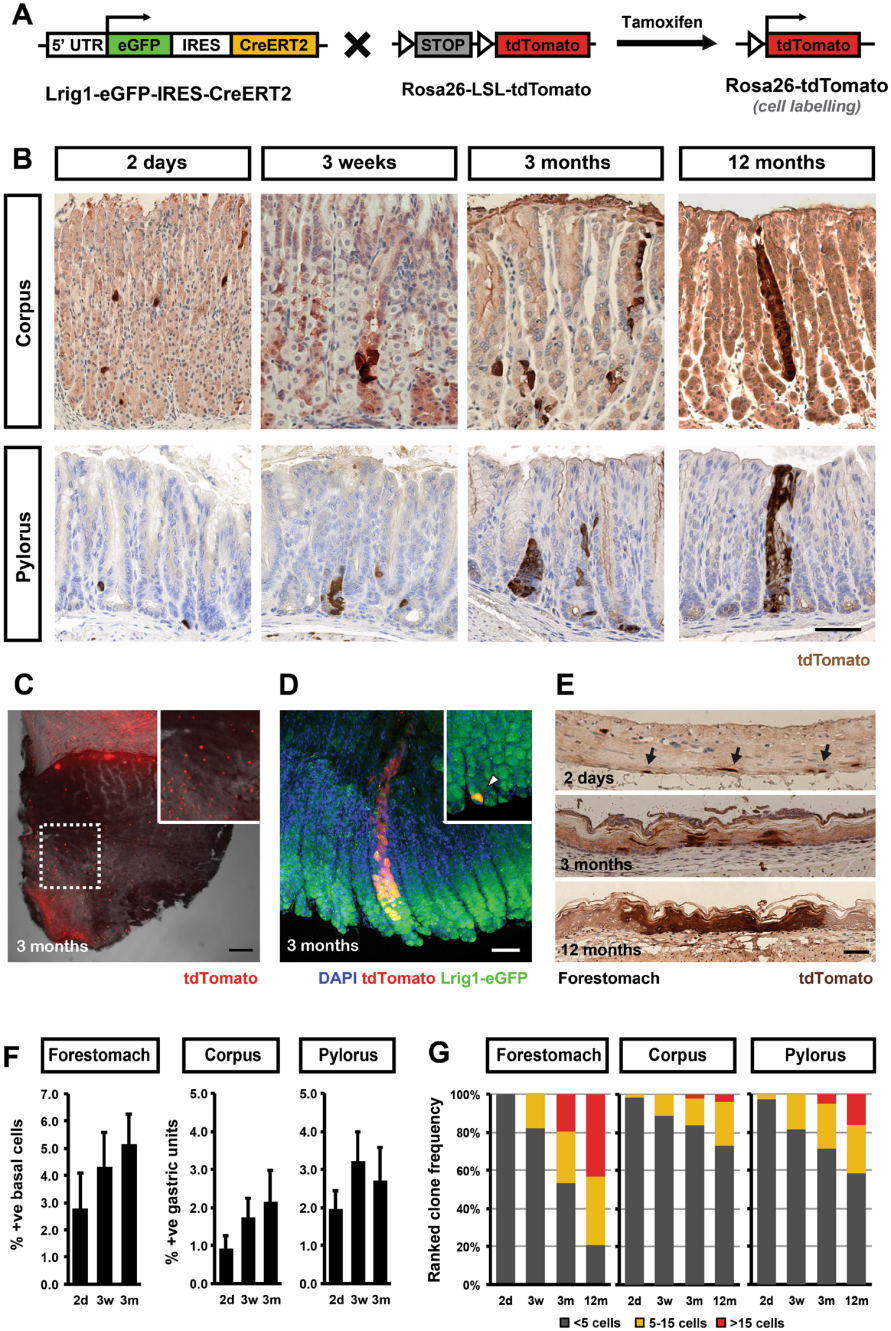
Fate mapping of Lrig1-expressing cells and their progeny. (**A**) Schematic representation of breeding strategy used to obtain inducible reporter mouse line used for tracing the lineage trajectory from Lrig1-expressing cells. LSL – loxP-Stop-loxP element; white triangles – loxP sites. (**B**) Analysis of clonal expansion of Lrig1-expressing cells in glandular gastric epithelium at the indicated time-points after induction with 100 μg of tamoxifen. Counterstaining with haematoxylin. (**C**) Whole-mount of mouse stomach analysed 3 months post induction with 2 mg tamoxifen showing extensive endogenous fluorescence of tdTomato in the forestomach and less ubiquitous clones in the glandular epithelium. (**D**) Different patterns of tdTomato-expressing clones included partially labelled glands and single cells, which did not divide at 3 months post labelling (arrowhead in the insert) with 100 μg of tamoxifen. (**E**) Analysis of clonal expansion of tdTomato-expressing cells in multi-layered stratified epithelium of the mouse forestomach at different time-points post labelling with 100 μg of tamoxifen. (**F**) Prevalence of tdTomato + cells at different time points post labelling as percentage of all basal cells (forestomach) or all gastric units (corpus and pylorus). (**G**) Size distribution of tdTomato + clones counted at different time points post injection. Scale bars: (**B**,**C**,**E**) −50 μm, C −2 mm.

In order to investigate the behaviour of Lrig1-expressing cells and their progeny in the gastric epithelium, we performed fate-mapping experiments using the Rosa26-*loxP*-STOP-*loxP*-tdTomato mouse model^15^. Offspring expressing Lrig1 KI was treated with tamoxifen to permanently mark Lrig1 expressing cells with tdTomato (Fig. 3A). The fluorescent mark inherited by daughter cells allowed us to follow the contribution of labelled cells over time and thereby assess their involvement in tissue maintenance (Fig. 3B). Whole-mount analysis of the stomach isolated from animals that received a single high dose of tamoxifen (2 mg) revealed that more or less the entire epithelium in the proximal part of the small intestine (duodenum) and most of the epithelium in the forestomach were labelled following a 3-month chase (Fig. 3C). In contrast, relatively few labelled glands were visible within the pylorus and corpus (Fig. 3C). In order to obtain clonal resolution of the cellular labelling and avoid the reported effects of cellular atrophy caused by high dose tamoxifen^16,17^, we labelled cells using a single application of 100 μg of tamoxifen. This induced long-term labelling of cells in 1–2% of the gastric units in the corpus and pylorus as well as 2–3% of the basal cells in the forestomach (Fig. 3B, E-G).

In line with the observations from the whole-mount experiment, analysis of multiple time points from fate mapping experiments performed at clonal level confirmed the participation of Lrig1-expressing cells in tissue maintenance (Fig. 3B). In the corpus and pylorus, we initially observed single labelled cells located in the lower part of the glands. After about 3 weeks their expansion lead to formation of groups of cells within individual glands. Some of these larger clones were subsequently maintained as clonal glandular units (Fig. 3B–G).

The behaviour of the labelled Lrig1-expressing cells was somewhat similar in the forestomach, where a larger fraction of clones was maintained over time and contributed to both to the basal layer and suprabasal differentiated layers (Fig. 3E). Overall, our analysis demonstrated that a subset of Lrig1-expressing cells in both corpus and pylorus as well as in the basal layer of the forestomach can be recruited to contribute to long-term tissue maintenance.

### Glandular gastric epithelial cells expressing Lrig1 have *in vitro* self-renewal capacity

**Figure 4:**
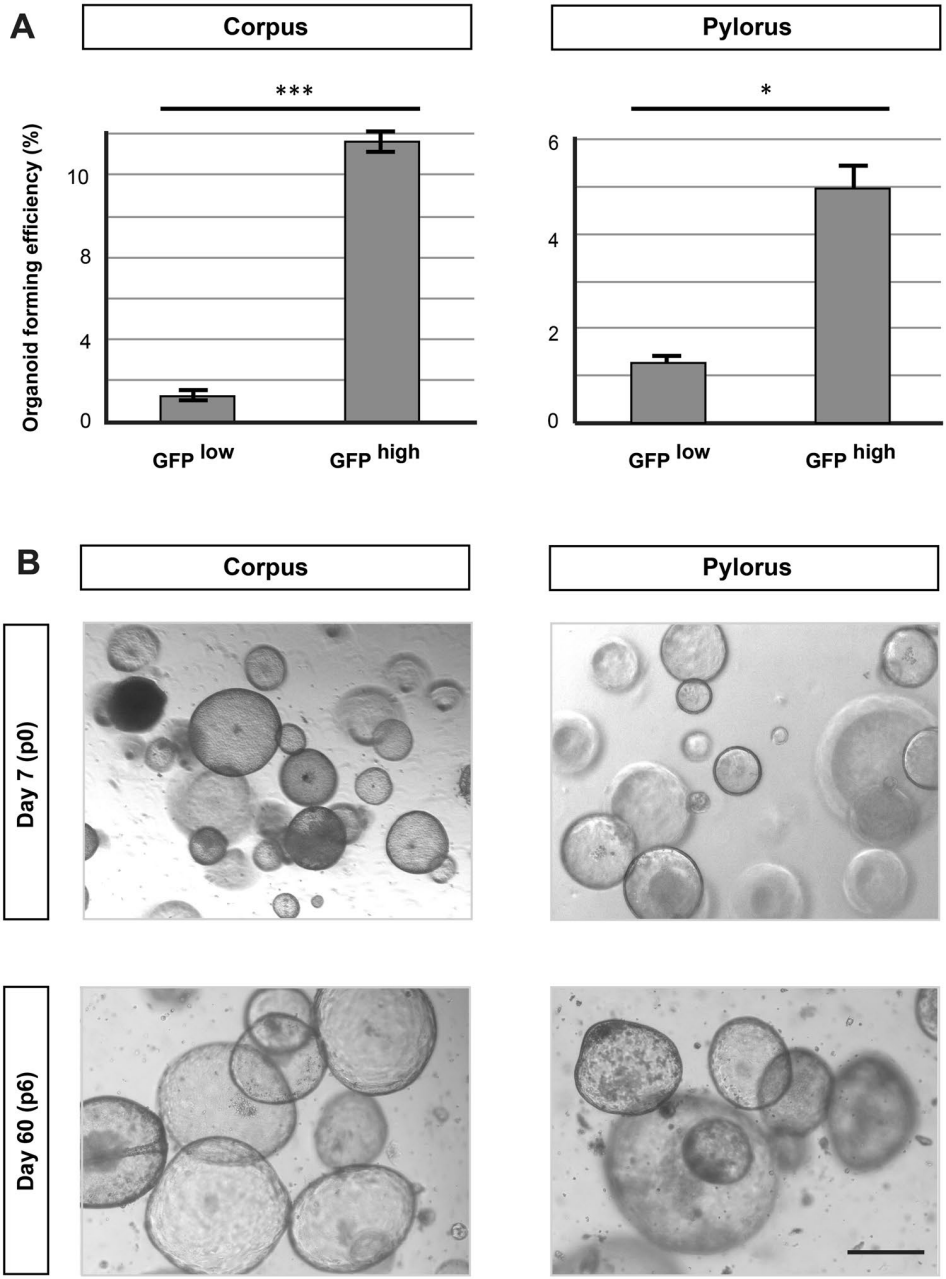
Analysis of *in vitro* clonogenic potential of Lrig1-expressing cells. (**A**) Comparison of organoid forming efficiency of *Lrig1*-eGFP low and *Lrig1*-eGFP high cells derived from stomach corpus and pylorus. *P ≤ 0.05, ***P ≤ 0.001. (**B**) Organoids derived from *Lrig1*-eGFP^high^ cells isolated from corpus and pylorus at 9 days (passage 0) and 60 days (passage 6). Scale bars −50 μm.

Previous reports revealed that cells from the gastric epithelium can be maintained *in vitro* using defined cell culture conditions^3^. These includes Troy-positive cells from the corpus^4^, and cells expressing Lgr5^3^ and Mist1^18^ as well as cells with Runx1 enhancer activity from both the corpus and pylorus^19^. In order to characterise the growth potential of Lrig1-expressing cells from the different parts of the glandular epithelium of the stomach, we analysed single cells isolated from the glandular compartment. Single cells were isolated using flow cytometry based on the expression of *Lrig1*-GFP (Fig. S1). Sorted *Lrig1*-GFP^high^ cells readily formed organoids *in vitro* with efficiencies similar to that previously reported for Lgr5-expressing cells from the pylorus and Troy-expressing cells from the corpus^3,4^. Importantly, the efficiency was much higher for *Lrig1-*GFP^high^ cells when compared to *Lrig1-*GFP^low^ cells (Fig. 4A), and gastric organoids derived form *Lrig1-*GFP^high^ population could be maintained long-term in defined medium without significant changes in morphology (Fig. 4B).

We conclude that a large proportion of *Lrig1* expressing cells in the glandular epithelium have *in vitro* self-renewal capacity.

## Discussion

A prerequisite for understanding the development and progression of diseases is insight into how tissues are maintained during normal tissue homeostasis. The stomach has been subject of much attention due to its important functions in digestion leading to diverging models for tissue maintenance^3,4,18–22^. Current observations converge upon a model, where cells at the gland base constitute a stem cell reservoir which become activated through cellular reprogramming/dedifferentiation following injury or genetic mutations^23,24^. It is tempting to speculate that the dedifferentiation is directed by changes in the local microenvironment, as recently described for the colonic epithelium^25^. Similarly, *in vitro* culturing using specified factors emphasise this change in cellular behaviour given that a large proportion of Lrig1-expressing cells have *in vitro* self-renewal potential.

Initial studies identified cells in the isthmus region as prospective stem cells of the gastric epithelium based on their migration and proliferative behaviour^26^. However, subsequent fate mapping studies have revealed the existence of alternative sources of cellular replenishment in the gastric epithelium. Here, cells located at the bottom of glands in the corpus region of the stomach have the capacity to contribute to homeostasis^3^, whereas TFF2^+^ cells constitute a transient source of replenishment, and chief cells expressing Mist1 and Gif contribute long-term in the remaining parts of the stomach^21,22^. The identity or identities of stem cells and progenitors involved in tissue maintenance of the stomach is consequently still unresolved, and it is unclear whether the proposed models are mutually exclusive, or whether contribution from seemingly differentiated cell types reflects injury induced reprogramming.

It is interesting that Lrig1 expression in the stomach epithelium was associated with the gland base, as this illustrated that a population of cells that has been believed to be lineage restricted can indeed be activated to contribute to the long-term to tissue homeostasis and that cell fate was indeed very plastic. Similar observations have recently been reported for the intestinal epithelium, where secretory and enterocyte progenitors can be reprogrammed into a stem cell like state upon tissue damage^27,28^. It remains a possibility that the observed plasticity in the intestine, described originally in regenerating tissues following severe damage, is a natural part of homeostasis in the gastric epithelium, where the stomach lumen provides a much more hostile environment.

The observation that Lrig1-expressing cells have the potential to contribute to long-term maintenance confirmed the existence of alternative mechanisms that are not dependent on cells in the isthmus region. This is in line with the previously reported reserve stem cell capacity identified within the chief cell compartment^21^. Moreover, the observed co-expression of Lrig1 and Lgr5, as well as *Lgr*5 and *Troy* enrichments in cells expressing Lrig1 isolated from small intestine, colon and now gastric glands suggested that similar environments is likely to support progenitor behaviour in the stomach and intestine. However, the overall composition of the niche is different judging from the proliferative behaviour of resident cells. It is worth noting that both Troy and Lgr5 are targets of Wnt-mediated signalling within the intestinal epithelium indicating that this is one potential common regulator between the different stem cell niches^29,30^. In contrast, Lrig1 is not known as a direct target gene of β-catenin/TCF following Wnt stimulation, but instead constitutes a target of cMyc, which is upregulated downstream of Wnt stimulation in other tissues^9,31^. Comparative analysis of the different niches, where Lrig1 is expressed is likely to shed additional light on whether Lrig1 expression reflects a particular cellular state or rather a unique microenvironment.

Results reported as a part of a previous larger study^32^ indicate that Lrig1-expressing cells are able to contribute to formation of Tuft cells, suggesting their role in tissue maintenance. While this manuscript was under consideration, another study by Choi *et al*. reported on the role of Lrig1-expressing cells in the gastric epithelium^33^. Using a different Lrig1-Cre mouse model than the one used in this study, the authors showed that Lrig1-expressing cells were indeed able to contribute to long-term tissue maintenance of the gastric epithelium. The effect was significantly enhanced during regeneration after a chemically-induced injury. Choi *et al*. proposed that Lrig1-expressing cells are a subtype of parietal cells in the neck region of gastric glands. In contrast, the evidence in this study demonstrates that Lrig1 is expressed by a heterogeneous population of cells not limited to the neck region, and comprising numerous cell types, including most notably parietal cells, but also chief cells.

In conclusion, we have demonstrated that a heterogeneous population of cells in the gastric epithelium expresses Lrig1 and that a small proportion of these have the potential to be activated and contribute to long-term tissue maintenance. Moreover, the ability of Lrig1-expressing cells to be activated to self-renew were evident, when cultured *in vitro* where these were highly enriched for cells with organoid forming capacity. Following characterisation of Lrig1 expression cells in the epidermis, small intestine and colon, this represents the fourth organ where Lrig1 expression is associated with cells that can contribute to the long-term tissue maintenance. It will consequently be important to address whether in other tissues Lrig1 expression similarly marks cell populations with stem cell and progenitor potential. Moreover, understanding how the gene is regulated will not only provide insight into how the different stem cell niches are maintained at the molecular level, but also how stem cell proliferation is controlled. The observation that in the gastric epithelium cells located towards the gland bottom can dedifferentiate and subsequently participate in tissue replenishment provides insights into how the lining of the stomach is maintained and lends additional support to a model where multiple cell populations at any given time have the capacity to contribute to long-term tissue maintenance in a context dependence manner.

## Methods

### Targeting of embryonic stem cells

The plasmid containing Lgr5-eGFP-IRES-CreER^T2^ was kindly provided by Hans Clevers. A unique NotI restriction site at the 3′ end of the eGFP-IRES-CreER^T2^ cassette was used to introduce a *loxP*-PGK-gb2-neo-*loxP* selection cassette (Gene Bridges ID: A003). Then a PCR product of the eGFP-IRES-CreER^T2^-*loxP*-PGK-gb2-neo-*loxP* was amplified with 50 bp homology arms corresponding to the Lrig1 genomic sequence flanking the knock in site. Using the Red/ET bacterial cloning system (Gene Bridges GmbH, Germany) this PCR product was cloned into a long genomic DNA fragment of Lrig1 which was subcloned from a BAC clone (bMQ mouse BAC library 129S7AB2.2 from Geneservice). This final knock-in construct was linearised and electroporated into C57Bl6 embryonic stem cells. Recombined ES clones were selected by Neomycin selection and screened by long-range PCR from homologous recombination. The PCR assays checked both the 5′ and 3′ region of the knock in sites by using internal/external primer pairs recognizing the eGFP-IRES-CreER^T2^-*loxP*-PGK-gb2-neo-*loxP* cassette (internal primers) and the genomic DNA sequence outside the homology arms (external primers). The selected clone was microinjected into blastocysts and the resultant chimeras were used to establish the Lrig1::eGFP-IRES-CreER^T2^-*loxP*-PGK-gb2-neo-*loxP* mice. The PGK-gb2-neo element was subsequently deleted by crossing with ubiquitously expressed Cre line (PGK-Cre)^34^ to obtain the Lrig1::eGFP-IRES-CreER^T2^ mouse line.

### Animal experiments

All *in vivo* experiments were performed in accordance with relevant guidelines and regulations under the terms of local regulations and supervision of suitable agencies. The National animal ethics committees in Denmark reviewed and approved all animal experiments. Rosa-CAG-LSL-tdTomato (Jax stock number 007905), has been described previously^15^. Lineage tracing experiments were induced by intraperitoneal injections of 2 mg or 100 µg of tamoxifen in 150 µl of corn oil. For DNA labelling experiments, mice received single intraperitoneal injection of EdU (50 mg/kg) and tissues were harvested after 30 min. After harvesting all tissues were flushed with PBS and fixed in 4% PFA for 24 h.

### Immunofluorescence, Immunohistochemistry and Imaging

For lineage tracing experiments, tissues were processed into FFPE blocks and 6 μm sections were cut. For all other experiments, tissues were processed as OCT frozen blocks and 9 μm sections were cut. Immunohistochemistry was performed using the ImmPRESS R.T.U. polymer detection kit (Vector Labs) or biotin-labelled secondary antibodies (Jackson ImmunoResearch) and streptavidin-HRP (Vector Labs). Immunofluorescence staining was carried out on frozen sections blocked in 0.5% BSA, 0.5% FSG and 0.1% Triton X100 before overnight incubation with primary antibodies. The following antibodies were used: anti-Lrig1 (R&D, AF3688 1:100), anti-GFP (AbCam, sb13970 1:500), anti-K14 (Covance, PRB-155P), anti-K10 (Covance, PRB-159P 1:100), anti-ATP4a (Millipore, 119101 1:300), anti-ChgA (Santa Cruz Biotechnology, sc-1488 1:100), anti-RFP/tdTomato (Rockland, 600-401-378 1:1000). Alexa-488, −555, and −647-conjugated secondary antibodies were from Thermo Fisher (1:400). *Lrig1*-eGFP and *Lgr5*-GFP in all figures represent immunostaining using anti-GFP antibody. For EdU incorporation experiments, a commercial kit allowing utilizing Alexa-647 fluorophore was used (Thermo Fisher, C10419). Nuclei were counterstained using DAPI or haematoxylin. Samples were imaged using Zeis Axio Observer (brightfield, fluorescent microscopy) or Leica SP5/8 TCS (confocal microscopy). In cases where Z-stacks were generated, maximum projection images were generated using ImageJ software or microscope manufacturer’s software. Stomach glands were isolated as described previously^35^, fixed in 1% PFA and imaged using confocal microscopy. Whole mounts were prepared from tissue flushed with ice-cold PBS, which was cut along the longitudinal axis and stretched and fixed in 4% PFA. Brightfield images and native tdTomato fluorescence were acquired using Leica Stereoscope. Overlay projection were generated using ImageJ software.

### Cell cytometry

For FACS analysis, mouse stomachs were washed extensively with PBS and carefully dissected. Forestomachs were discarded and glandular compartments (corpus and pylorus) were cut into small pieces (ca. 3 mm). Tissue fragments were chelated in an EDTA-containing buffer (10 mM) as described previously^35^. Separated epithelium was digested using Dispase II (1U/ml Roche, 04-942-078-001) for 10 min. at 37 °C. After filtration cells were stained using the following antibodies: anti-Epcam-APC (BD 347200), anti-CD45-PE-Cy7 (BD 552848), anti-CD31-PE-Cy7 (eBioscience 25-0311-82) for 30 min. on ice, washed in PBS supplemented with 1% BSA and sorted using FACS Aria I/III (BD). CD45^+^ and CD31^+^ cells were excluded. Results were analysed using the FlowJo software package.

### Expression analysis

Cells isolated using FACS were immediately transferred into cell lysis buffer and processed using an RNA isolation kit (Ambion, 12183018A). cDNA was synthetised utilising SuperScript III oligo-dT and random hexamers (Thermo Fisher, 18080044). Gene expression was measured by qPCR using SYBR Green PCR Master Mix (LifeTechnologies, 4385612) and the following primer pairs (5′−3′): *Lrig1* (Fw: ttgaggacttgacgaatctgc, Rev: cttgttgtgctgcaaaaagagag), *Ki67* (Fw: atcattgaccgctcctttaggt, Rev: gctcgccttgatggttcct), *Tff1* (Fw: agcacaaggtgatctgtgtcc, Rev: ggaagccacaatttatcctctcc), *Tff2* (Fw: acccgggcatcagtcccga, Rev: gcagctcccagggaacgggt), *Muc5a* (Fw: gtggtttgacactgacttccc, Rev: ctcctctcggtgacagagtct), *Pgc* (Fw: tgcctaccctcacttttgtcc, Rev: cactctcagcgttcagggag), *Gif* (Fw: cctggggccttattgtctcttc, Rev: tgaagttggctgtgatgtgc), *Atp4a* (Fw: tctgctttgcgggacttgta, Rev: cggcatttgagcacagcat), *Lgr5* (Fw: acccgccagtctcctacatc, Rev: gcatctaggcgcagggattg), *Gast* (Fw: acacaacagccaactattc, Rev: caaagtccatccatccgtag), *Gapdh* (Fw: ggtgaaggtcggtgtgaacg, Rev: ctcgctcctggaagatggtg). Reactions were run on StepOne (AppliedBiosystems) or QuantStudio (LifeTechnologies) machines using default program parameters. Each PCR reaction was carried out in triplicate and the relative quantification of gene expression analyzed using the ΔΔCT method with *Gapdh* as the endogenous reference.

### Cell culture

Single cells isolated using flow cytometry were seeded in 25 μl of Matrigel mixed with recombinant Jagged-1. 1000 or 3000 cells were seeded into a single well of a 48-well plate. Cells were cultured in defined Advanced DMEM/F12 medium (Life Technologies, 12634010) containing 1x penicillin/ streptomycin (Life Tehcnologes, 15140122), 10 mM HEPES (Gibco, 15630080), 2 mM GlutaMAX (Gibco, 35050061), 10 μM Y-27632, 500 ng/ml mRspondin-1 (R&D), 100 ng/ml mNoggin (R&D), 50 ng/ml hEGF (Peprotech), 10 nM gastrin (Sigma), 100 ng/ml hFGF10 (Peprotech) 3 μM CHIR99021 (Calbiochem). Cultures were maintained in a 37 °C incubator with constant atmosphere of 5% CO_2_. Cells were seeded in triplicate wells. Medium was changed every 2–3 days. After 7 days all epithelial organoids present were counted manually based on images acquired with a Leica Microscope (DMIL LED). For further expansion of gastric organoids (passages 1–6), CHIR99021 and Y-27632 were omitted. Organoids were split mechanically by pipetting vigorously 30 times with a P200 every 7–9 days.

### Quantitation and Statistical analysis

Quantitation of Lrig1 expression along the glandular axis was carried out using the plot profile analysis functions of ImageJ in at least 50 regions using three tissue sections. In order to assess labelling efficiency, 400 glands from two independent tissue sections and for three biological replicates have been counted. Statistical analyses were conducted using MS Excel (Microsoft), which was also used to prepare plots and charts. One-tailed, unpaired Student’s t test was utilised, with p < 0.05 considered statistically significant. Error bars represent the standard deviation.

## Electronic supplementary material

Supplementary figure
