# Supplementary material for: Lrig1 marks a population of gastric epithelial cells capable of long-term tissue maintenance and growth in vitro
Source: Sci Rep. 2018 Oct 15;8:15255. doi: 10.1038/s41598-018-33578-6 (PMC6189208; doi:10.1038/s41598-018-33578-6)
Supplement: Supplementary file 1 — Supplementary figure [file 41598_2018_33578_MOESM1_ESM.pdf]

Lrig1 marks a population of gastric epithelial cells capable of long-term tissue maintenance and growth *in vitro*

Pawel J. Schweiger, Ditte L. Clement, Mahalia E. Page, Troels Schepeler, Xiangang Zou, Gabor Sirokmány, Fiona M. Watt & Kim B. Jensen

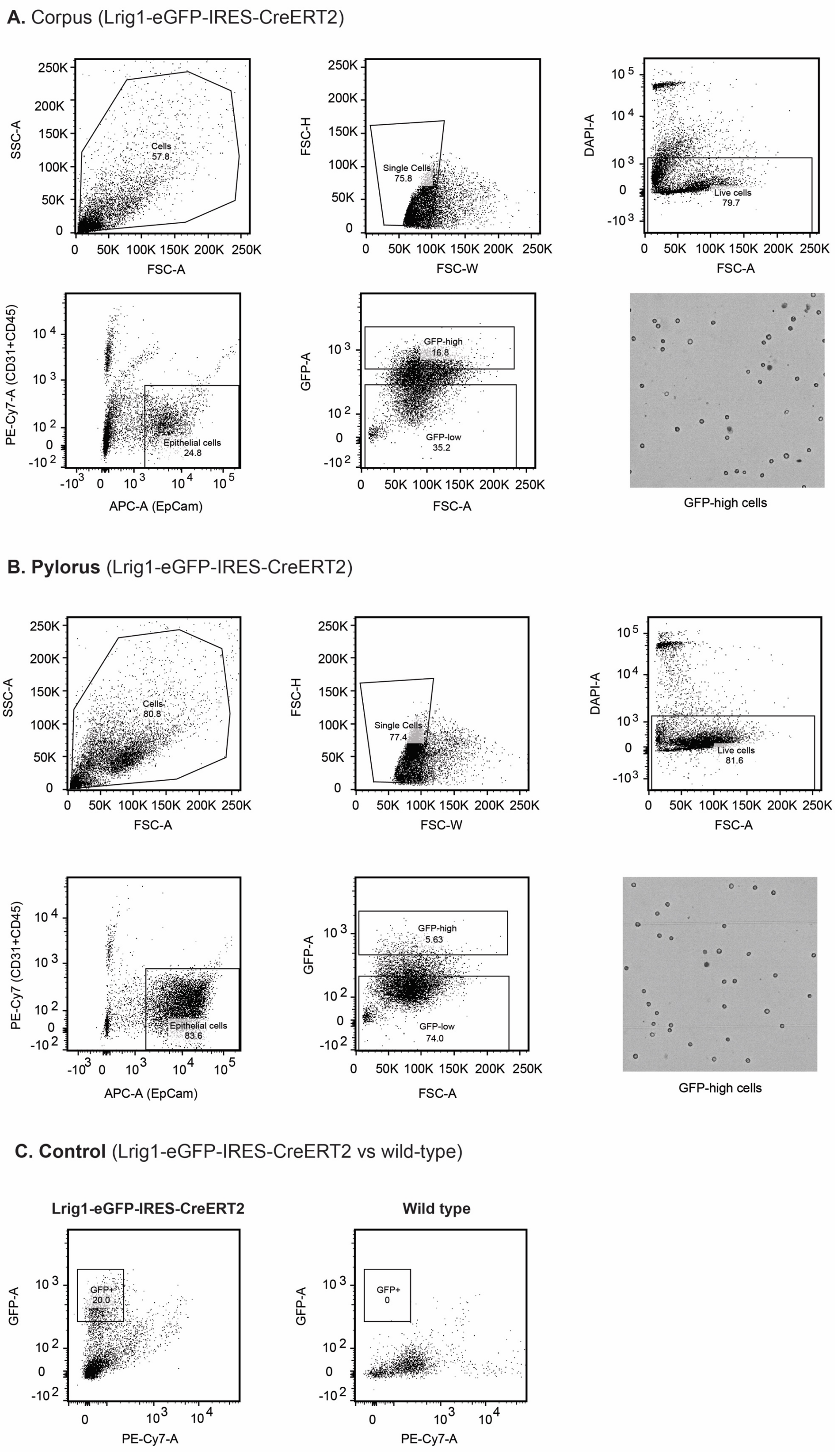

Figure S1. Strategy for FACS isolation of Lrig1^{high} cells from mouse stomach

(A,B) Gating strategy for isolation of cells expressing different levels of *Lrig1*-eGFP from the stomach corpus and pylorus of Lrig-KI mice. Pictures of cells post FACS confirming the successful isolation of single cells. (C) Comparison of fluorescence in the green channel between the Lrig1-KI mice and a wild-type control indicating the extent of the shift of the Lrig1^{high} population
